# Are time-trends of smoking among pregnant immigrant women in Sweden determined by cultural or socioeconomic factors?

**DOI:** 10.1186/1471-2458-10-374

**Published:** 2010-06-26

**Authors:** Kontie M Moussa, P-O Ostergren, Frida Eek, Anton E Kunst

**Affiliations:** 1 Department of Clinical Sciences Malmö, Division of Social Medicine and Global Health, Lund University, Malmö University Hospital, Malmö, Sweden; 2 Division of Occupational and Environmental Medicine, Lund University, Lund University Hospital, Lund, Sweden; 3 Department of Public Health, Academic Medical Centre (AMC), University of Amsterdam, the Netherlands

## Abstract

**Background:**

The widening socioeconomic gap in smoking during pregnancy remains a challenge to the Swedish antenatal care services. However, the influence of cultural factors in explaining the socioeconomic differences in smoking during pregnancy is not clear among the immigrant women. The aim of this study was to investigate whether the development of smoking prevalence among pregnant immigrant women in Sweden followed the trajectory which could be expected from the stages of the global smoking epidemic model in the women's countries of origin, or not.

**Methods:**

Delivery data on pregnancies in Sweden from 1982 to 2001 was collected from the Swedish Medical Birth Registry. From a total of 2,224,469 pregnant women during this period, all immigrant pregnant women (n = 234,731) were selected to this study. A logistic regression analysis and attributable fraction were used to investigate the association between smoking during pregnancy and the socioeconomic differences among immigrant women.

**Results:**

Overall, the prevalence of smoking among pregnant immigrant women decreased from 30.3% in 1982 to 11.0% in 2001, albeit with remarkable differences between educational levels and country of origin. The greatest decline of absolute prevalence was recorded among low educated women (27,9%) and among other Nordic countries (17,9%). In relative terms, smoking inequalities increased between educational levels regardless of country of origin. The odds ratios for low educational level for women from other Nordic countries increased from 4.9 (95% CI 4.4-5.4) in 1982 to 13.4 (95% CI 11.2-16.2) in 2001, as compared to women with high education in the same group. Further, the total attributable fraction for educational difference increased from 55% in 1982 to 62% in 2001, demonstrating the strong effect of educational attainment.

**Conclusions:**

Our hypothesis that the socioeconomic time trend of smoking based on the stage of the world wide tobacco epidemic model related to country of origin of the immigrant women was not supported by our analyses. Our findings does not support a call for specific "culture sensitive" antismoking policies or interventions in Sweden or similar countries, but reinforce the existing evidence with a focus on women with a low educational level, regardless of cultural background.

## Background

Smoking during pregnancy has been identified as a major risk factor both for future health of the woman and of the fetus/child [1], which gained a considerable public health attention in Sweden. As a result, an intensive anti-tobacco intervention in antenatal care services were launched, which in turn contributed to a significant decline of smoking prevalence among pregnant women. However this decline was more rapid among women with higher socioeconomic status, and therewith widening social inequalities in smoking in Sweden was inevitable [2].

Women with low socioeconomic status include immigrants, lone mothers and young women, who remain as a major challenge to the tobacco interventions in the country. It has been debated whether there is a specific need for culturally tailored programmes, in order to reach pregnant immigrants, who now contribute to more than 20% of yearly deliveries in Sweden.

During the last five decades Sweden has experienced several waves of immigration. The immigration patterns varied from work force migration in the 1960s and 1970s, mostly from other Nordic and other European countries, to a growing proportion of asylum seekers and humanitarian refugees in late 1990s, now also from non-European countries [3]. Stress arising from displacement and introduction into new environment has been shown to expose immigrants to excess health risks and potential health hazards [4]. Consequently, both low socioeconomic- and immigrant status are associated with the risks of less benefit to public health interventions, which in turn contribute to widening health gap, [5-8] and smoking has been identified as an important factor behind these inequalities [2,9].

Longitudinal data on the development over time of the socioeconomic pattern of smoking in pregnant immigrant women is sparse in the scientific literature and the few available studies indicate the presence of behaviour differences in smoking during pregnancy between immigrant and native women in UK [10] and USA [11]. Other studies have reported that immigrant women from low income countries bring with them beliefs, traditions, and cultural practices of their home countries such as negative attitudes towards smoking among women [12]. However, after longer stay in their new countries with greater access to tobacco and fewer normative restrictions, immigrant women may adopt the smoking habits of their host countries [11,13,14]. A longitudinal approach that sheds light on the socioeconomic and cultural impact on smoking habits would be of great relevance for understanding the development of health inequalities [1,15-17].

The tendency to respond to health messages during pregnancy may differ due to socio-cultural backgrounds and to the stage of the global tobacco epidemic in the countries of origin of the women. The global tobacco epidemic model was originally based on the progression of cigarette smoking and smoking induced mortality as a general phenomenon and according to the socioeconomic patterning. The model thereby describes different stages of the epidemic [18]. In the first stage of the epidemic, the smoking prevalence increases among men especially in higher socioeconomic strata but remains at low levels among all women. Most of countries in sub-Saharan Africa fall to this stage. In the second stage, smoking continues to increase among all socioeconomic levels of men reaching a peak and now there is also a rapid increase among women in high socioeconomic position. The concerned countries in this stage are: countries in South East Asia, North Africa, Latin America as well as Japan and China. The third stage of the tobacco epidemic model includes countries in Southern and Eastern Europe, where smoking prevalence is high in both sexes but start to decline rapidly among men in higher socioeconomic strata. Moreover, the prevalence starts to decrease even among women with high education but slowly compared to men. In the fourth phase, smoking decreases in slower fashion among both men and women, however more in men and more in higher socioeconomic strata. Countries in Western Europe, USA, Canada and Australia appear in stage four of the epidemic. During this phase, smoking prevalence will remain high among those in low socioeconomic strata, and thus there is a situation of growing inequalities in this major health determinant [19-21].

Therefore, a comparison between time-trends of smoking among pregnant immigrant women in Sweden and the same trends in the general population may provide useful information for the evaluation of the impact of the Swedish anti-tobacco policies in the two mentioned groups. Since the general trends have been analyzed in a previous study[2], those findings need to be supplemented with a specific analysis regarding the pregnant immigrant women during the same time period.

The aim of this study was to investigate whether the development of the smoking prevalence at the first antenatal visit in pregnant immigrant women in Sweden during the period 1982-2001, and the socioeconomic distribution of smoking in this group, followed the trajectory which could be expected from the stages of the global smoking epidemic model in the women's countries of origin, or not.

## Methods

### Study population

Data on all pregnancies in Sweden resulting in a delivery from 1982 to 2001 was collected from the Swedish Medical Birth Registry (MBR), which preserves health profiles, marital status, age, nationality, and other demographic factors for all pregnant women in Sweden who participate in the national health system [22]. Our total study cohort consisted of 2,224,469 individuals. Out of these 234,731 women born in other countries than Sweden were targeted for this study. A similar analysis based on the same data source has been described in detail elsewhere [2].

### Definitions

Smoking status at first antenatal care visit was categorised and assessed as follows: 1) Non-smoker, 2) smoke one to nine cigarettes per day, and 3) smoke ten or more cigarettes per day.

*Outcome variable: *A woman who smoked at least one cigarette per day at the time of her first antenatal visit was classified as a smoker.

### Exposure variables

*Country of origin *options were, either born in a) Other Nordic countries (Denmark, Finland, Iceland and Norway), b) Other European countries, or c) Non-European countries.

The *education variable *was coded into three levels of education, according to number of years a person was enrolled in school, i.e., low educational level (up to nine years), middle educational level (10 to 12 years), and high educational level (more than 12 years).

*Marital status: *Married women or those women co-habiting with a partner were classified as married; other pregnant women were classified as single mothers.

*Age *was categorised into three ranges: 16-24 years, 25 to 34 years, and 35-44 years. In our database the age of pregnant women ranged from 16-44 years.

### Statistical analyses

We began by using prevalence as the measure for comparisons over time between the groups studied in order to capture tendencies on an absolute level. Next, multiple logistic regression analyses were performed, yielding odds ratios based on prevalences. In order to eliminate potential confounding from age and marital status, a step-wise multiple logistic regression analysis was also performed, yielding adjusted odds ratios. Prevalences and odds ratios (OR) were calculated using SPSS software Version 16.0 [23].

The attributable fraction (AF) was calculated using the formula: AF = (OR - 1)/OR, where OR is the adjusted odds ratio generated by multiple logistic regression analysis [24]. Total attributable fraction (TAF) was calculated as follows:

where AF_i _is the attributable fraction for smoking at first antenatal visit for a specific stratum (here: educational level), and P _i_represents the proportion of all cases that fall in this stratum. The expression within the parenthesis thus represents the stratum-specific total attributable fraction (sTAF), and ∑ indicates the summation of all the strata-specific calculations, which in turn results in the overall TAF. For those with the highest level of education, the AF and sTAF are by definition zero [25]. Finally, overall TAF is the summation of the sTAFs and represents the proportion of smoking that would not exist if all pregnant women had had the same prevalence as those with highest level of education, under the assumption that there is a causal pathway between educational level and the outcome variable.

## Results

The demographic characteristics of immigrant pregnant women in our study are provided in table 1. The smoking prevalence during pregnancy in Sweden decreased from 30.3% in 1982 to 11.0% in 2001. Women from other Nordic countries were a great majority (55.2%) in 1986; however this group shrank to 14.7% in 2001. Oppositely, the proportion of women from Non-European countries increased from 18.6% in 1982 to 56.1% in 2001, while the proportion remained constant for women from European countries. During the period studied, the proportion of women with low-, middle- and high educational level was 28%, 46%, and 26%, respectively. The large majority of women were in age group of 25-34 years, married or cohabiting with a partner (Table 1).

**Table 1:** Demographic description of pregnant foreign-born (migrant) women at first visit at antenatal clinic from 1982-2001 and valid percent (%) presented in 5 years intervals.

	**1982-1986**		**1987-1991**		**1992-1996**		**1997-2001**	
	**n**	**%**	**n**	**%**	**n**	**%**	**n**	**%**
	
Smoking status								-
Smokers	12081	30.3	13440	25.6	9545	17.4	7133	11.0
None-smokers	27727	69.7	39118	74.4	45245	82.6	57862	89.0
Missing	8842	--	4849	--	3144	--	5745	--
Total	48650		57407		57934		70740	
*Education*								
Low level	7 916	28.9	12 238	29.9	12431	26.0	16799	27.3
Middle level	13 140	48.0	19 867	48.3	22560	47.1	25919	42.1
High level	6 320	23.1	9 026	21.9	12912	27.0	18858	30.6
Missing data	21 274		16 276		10031		9164	
Total	48650		57407		57934		70740	
*Age group*								
16-24 years	13349	27.4	13858	24.1	11901	20.5	14849	21.0
25-34 years	28701	59.0	34784	60.6	35931	62.0	42621	60.3
35- years	6600	13.6	8765	15.3	10102	17.4	13270	18.8
Missing data	0		0		0		0	
Total	48650		57407		57934		70740	
*Marital status*								
Married	39507	95.9	50208	95.4	49179	92.6	62024	94.5
Single	1697	4.1	2408	4.6	3929	7.4	2580	5.5
Missing data	7446		4791		4826		5136	
Total	48650		57407		57934		70740	
*Country of origin*								
Nordic countries	26864	55.2	26065	45.4	18310	31.6	10383	14.7
European countries	12747	26.2	15598	27.2	16123	27.8	20700	29.3
Non-European countries	9039	18.6	15744	27.4	23501	40.6	39657	56.1
Missing data	0		0		0		0	
Total	48650		57407		57934		70740	

(n = 234 731)

The prevalence of smoking at the time of the first antenatal visit among immigrant women decreased in all educational groups, but more so in the lower and middle categories, as compared with the highest. Subsequently, the smoking prevalence among pregnant women with high educational level has decreased from 16.1% to 4.6% (a prevalence difference of 11.5%), and for women with middle educational level the corresponding decrease was from 31.8% to 12.1% (a prevalence difference of 19.7%). However, the highest prevalence reduction was recorded by women with low educational level from 44.6% to 16.7% (a prevalence difference of 27.9%). Likewise, the decline of the prevalence among pregnant women from different countries 1982-2001 shows that this has decreased from 37.9% to 20.0% among women from other Nordic countries; from 26.4% to 16.9% among women from other European countries; and from 13.3% to 5.5% among women from Non-European countries. Thus, on an absolute scale smoking decreased less among women with highest educational level. Among the age groups, the highest absolute reduction in smoking prevalence was seen among women 16-24 years of age followed by those in age group 25-34 years compared to eldest group (24.7%, 18.8%, and 12.4%, respectively). Moreover, a considerable absolute reduction in smoking prevalence has been recorded among single mothers and women born in other Nordic countries. As a general pattern, the highest prevalence declines were seen in the groups that had the highest smoking rates at baseline 1982 and the socio-demographic disparities seemed to be narrowing on an absolute scale (Table 2).

**Table 2:** Number of pregnant immigrant smokers (n) and smoking prevalence (%) according to socio-demographic variables during first visit at antenatal clinic from 1982-2001 presented in 5 years intervals

	**1982-86**		**1987-1991**		**1992-1996**		**1997-2001**	
***Educational level***	**n**	**%**	**n**	**%**	**n**	**%**	**n**	**%**
	
High level	823	16.1	1044	12.5	1019	8.3	797	4.6
Middle level	3461	31.8	5076	27.8	4269	19.9	2889	12.1
Low level	2944	44.6	3905	35.1	3052	26.5	2587	16.7
Total	7228		10025		8340		6273	
*Age group*								
16-24 years	4022	37.0	3800	30.1	2092	18.6	1687	12.3
25-34 years	6725	28.7	7713	24.2	5672	16.7	4002	10.2
35- years	1334	24.4	1927	24.0	1781	18.7	1444	12.0
Total	12081		13440		9545		7133	
*Marital status*								
Married	10968	29.3	11569	24.4	7625	16.0	6176	10.3
Single	868	54.2	1113	48.9	1324	35.0	749	21.7
Total	11836		12682		8949		6925	
*Country of origin*								
Other Nordic countries	8392	37.9	8389	35.0	5005	28.8	1903	20.0
Other European countries	2703	26.4	3447	24.2	2791	18.4	3252	16.9
Non-European countries	986	13.3	1604	11.2	1749	7.9	1978	5.5
Total	12081		13440		9545		7133	

Table 3 shows the prevalence of smoking at each educational level according to country of origin and the crude odds ratios with 95% CI. The results demonstrate an increasing relative difference in odds ratios during the period studied between educational levels regardless of country of origin. The crude OR for pregnant women with low educational level from other Nordic countries than Sweden increased from 4.9 (4.4-5.4) in 1982 to 13.4 (11.2-16.2) in 2001, as compared to women with high education in the same group. During the period of 1982-2001, the crude ORs for smoking during pregnancy among women with low education from other European countries increased from 2.9 (2.4-3.6) to 4.0 (3.5-4.5) compared to their counterparts with high level of education. Furthermore, the crude ORs increased from 1.2 (1.6-2.8) to 3.4 (3.0-4.0) for women with low educational level from Non-European compared to those with high educational level (Table3). When adjusted for age and marital status the odds ratios between educational groups changed marginally (data not shown).

**Table 3:** Prevalence (%) of smoking during pregnancy at first antenatal care visit and crude odds ratios (OR) with 95% confidence intervals (CIs) for each five-year period according to educational levels and country of origin from 1982-2001.

	Other Nordic Countries		Other European Countries (Nordic countries excluded)		Non-Europeans Countries	
	**%**	**OR (95% CI)**	**%**	**OR (95% CI)**	**%**	**OR (95% CI)**
	
*1982-1986*						
High level	16.9	1.0	16.6	1.0	12.6	1.0
Middle level	36.1	2.8 (2.5-3.1)	27.8	1.9 (1.6-2.3)	16.4	1.4 (1.1-1.8)
Low level	49.7	4.9 (4.4-5.4)	36.9	2.9 (2.4-3.6)	23.4	2.1 (1.6-2.8)
*1987-1991*						
High level	14.0	1.0	13.8	1.0	7.5	1.0
Middle level	33.9	3.2 (2.9-3.5)	25.1	2.1 (1.8-2.4)	13.4	1.9 (1.6-2.3)
Low level	51.4	6.5 (5.9-7.2)	33.4	3.1 (2.7-3.7)	13.2	2.0 (1.6-3.7)
*1992-1996*						
High level	10.5	1.0	10.5	1.0	4.8	1.0
Middle level	28.6	3.4 (3.0-3.8)	19.1	2.0 (1.8-2.3)	9.8	2.1 (1.8-2.5)
Low level	49.6	8.4 (7.4-9.4)	25.4	2.9 (2.5-3.3)	10.3	2.3 (1.9-2.7)
*1997-2001*						
High level	5.6	1.0	7.8	1.0	2.5	1.0
Middle level	20.6	4.4 (3.7-5.2)	17.6	2.5 (2.2-2.8)	5.9	2.5 (2.1-2.9)
Low level	44.4	13.4 (11.2-16.2)	25.2	4.0 (3.5-4.5)	8.1	3.4 (3.0-4.0)

n = 234, 731

Table 4 shows the association between smoking at first antenatal visit and educational level, adjusted OR (for age marital status, and country of origin), AF, sTAF, and overall TAF between 1982 and 2001. A further illustration shows that sTAF for low educated women increased from 29% to 33% between the periods 1982 to 2001, indicating the widening gap in smoking between educational levels among immigrant women at the time of their first antenatal visit. Further, the total attributable fraction for educational differences increased from 55% in 1982 to 62% in 2001, demonstrating that the association with educational attainment regarding the studied type of smoking behaviour, increased somewhat during the studied period. For instance, the calculation of attributable fraction was demonstrated according to table 4. Attributable fraction (AF) = (OR-1)/OR; AF for low educational level 1982 -1986 would be (3.6-1/3.6 = 0.72), hence stratum specific total attributable fraction (sTAF) for that group can be calculated by multiplying the proportion of the group in that stratum by AF that yields; 0.407*0.72 = 0.29 or 29% (Table 4).

**Table 4:** Number of foreign-born smokers (n) during pregnancy and their proportion (%) in each educational level stratum, adjusted Odds Ratios (aOR)* and 95% Confidence Interval (CI) for smoking in those strata, and Attributable Fraction (AF), Stratum-specific Total Attributable Fraction (sTAF), and overall Total Attributable Fraction (TAF) for each five-year period.

**Educational levels**	**n**	**%**	aOR* (95% CI)	**AF**	**sTAF**	**TAF**
	
*1982-1986*						
High educational level	823	11.4	1.0	0 (Ref)		
Middle educational level	3461	47.9	2.2 (2.0-2.4)	0.55	0.26	
Low educational level	2944	40.7	3.6 (3.3-3.9)	0.72	0.29	
TAF, all levels of education						0.55
*1987-1991*						
High educational level	1044	10.4	1.0	0 (Ref)		
Middle educational level	5076	50.6	2.6 (2.4-2.8)	0.62	0.31	
Low educational level	3905	39.0	4.3 (3.9-4.7)	0.77	0.30	
TAF, all levels of education						0.61
*1992-1996*						
High educational level	1019	12.2	1.0	0 (Ref)		
Middle educational level	4269	51.2	2.5 (2.3-2.7)	0.60	0.31	
Low educational level	3052	36.6	4.1 (3.8-4.5)	0.76	0.28	
TAF, all levels of education						0.59
*1997-2001*						
High educational level	797	12.7	1.0	0 (Ref)		
Middle educational level	2889	46.1	2.8 (2.6-3.1)	0.64	0.29	
Low educational level	2587	42.2	4.8 (4.4-5.3)	0.79	0.33	
TAF, all levels of education						0.62
Total smokers 1982-2001	31866					

* adjusted for age and marital status.

(n = 31866)

**Figure 1:**
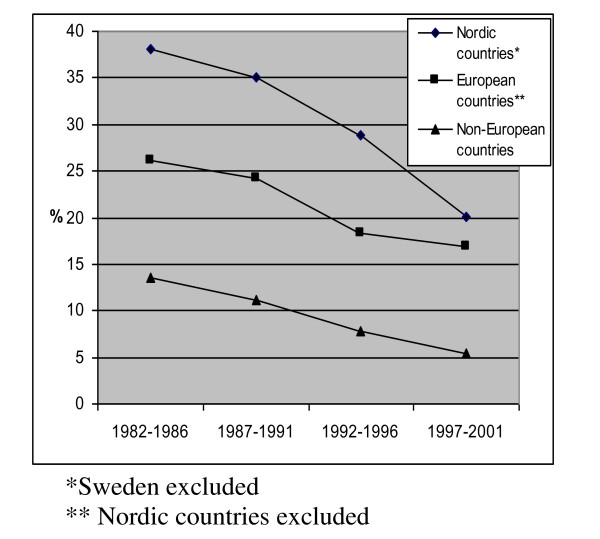
**Smoking prevalence during pregnancy among immigrant women in Sweden 1982-2001**.

Figure 1 illustrates trends in smoking prevalence among immigrant pregnant women in Sweden according to country of origin from 1982-2001. The overall trend shows that smoking during pregnancy has declined in all groups. However, the highest absolute reduction in prevalence was seen among women born in other Nordic countries with 18.0%, which also had highest prevalence of smoking 1982 compared to other groups. The gap of smoking prevalence between women born in Nordic and European countries is narrowing, however still remaining higher compared to women born in non-European countries (Figure 1).

## Discussion

The main findings of our study indicate that there has been a noteworthy overall decline in smoking prevalence among pregnant immigrant women in Sweden during the period studied. In absolute terms the smoking prevalence declined among all groups indicating reduced socioeconomic inequality. However, a relative increase of inequality was shown which persisted also after adjusting for age and marital status. The increasing socioeconomic differences were more evident among women born in Nordic countries. Among this group the adjusted ORs for low educational level increased from 4.9 (4.4-5.4) to 13.4 (11.2-16.2) during 1982-2001. The corresponding increase among women from non-European countries during the same period was from OR 2.1 (1.6-2.8) to 3.4 (3.0-4.0). The widening socioeconomic gap in smoking during pregnancy among women from other Nordic countries has been shown to be similar to the trend observed among Swedish pregnant women [2]. Our results show that the socioeconomic inequality in smoking widened between women born in Europe and women born in non-European countries. The observations do not support the assumption that smoking trends in the studied groups of immigrant women could be predicted by the world-wide tobacco epidemic model, i.e. by comparing with the socioeconomic trends in smoking among women in their particular countries of origin. According to this model, women with high education from non-European countries would be expected to have higher smoking prevalence compared to women from Nordic countries. In contrast, our findings show that women with high education smoke less during pregnancy, regardless of the stage of the tobacco epidemic of their countries of origin.

Thus, our original hypothesis must be rejected on basis of our findings and alternative explanations should be sought.

When considering the development of smoking patters in general in Sweden as well as the socioeconomic pattern of smoking of immigrants, it could be important to consider the process of acculturation. Berry's typology of acculturation strategies is based on the combination of two main factors, preservation of the culture of origin and degree of participation in the new country [26]. This will yield four situations of the immigrant population, marginalization (low degree of keeping the original culture and low degree of participation in the new society), isolation (high degree of keeping the original culture and low degree of participation), assimilation (low degree of keeping original culture and high degree of participation) and finally integration (high degree of keeping original culture and high degree of participation). Interpreting the development of smoking prevalence among pregnant immigrant women according to Berry's model indicate that our findings fit less with aspects of marginalization and isolation, but better with Berry's description of assimilation and integration. Hence, the trend of smoking habits among immigrant women seems to be shaped by the same influences as those which have affected the whole population, (e.g. general Swedish tobacco prevention initiatives on women's smoking). Thus, our findings strongly imply that the most important determinant of smoking trend among pregnant immigrant women in Sweden was associated with the level of education rather than women's cultural background.

An important factor to be taken into consideration, is the change over time regarding the geographic region of the country of origin of the immigrant women, namely from Europe (particularly other Nordic countries) in the beginning of the studied period, to a growing proportion of immigrants from non-European countries. In those countries smoking prevalence is usually low among all women, regardless of educational level, which ought to favour less socioeconomic inequality over time if they retained their original smoking habits, if the original hypothesis were true. This is also contrary to our analyses, since inequality did not decrease over time. This picture is also more compatible with our alternative hypothesis based on Berry's typologies of integration.

A third factor that might be considered is the role that 'healthy immigrant effect' plays in immigrants' health behaviour. The selection of healthy individuals in the migration process might contribute to that non-smokers actively would make a healthier choices to not initiate smoking, assuming that they originate from a society with lower tobacco consumption, where cultural and traditional values have a protective impact [27], in combination with Swedish tobacco control policy.

Strengths of this study are the size of the sample, the long study period, and its limitation to the first generation of immigrant women. Another strength is that data from the Swedish Birth Register is little sensitive to selection bias, since almost all deliveries are taking place in hospitals and are thereby registered.

Under-reporting of the outcome (smoking during first antenatal visit) has been a concern of validity with regard to self reported smoking. Several studies however suggest that self-reported data on smoking to be a good and reliable indicator of the daily smoking [28,29].

The limitations of this study include the difficulty to ascertain the evidence whether the women started or quit smoking in Sweden or in their countries of origin as well as the difficulty to interpret the concept of acculturation since we lack data on numbers of years lived in Sweden and age of immigration. Additionally, our data lacks more explanatory variables such as employment status and income.

## Conclusions

Our hypothesis that the socioeconomic time trend of smoking at first ante-natal visit based on the stage of the world wide tobacco epidemic model of the country of origin of the immigrant women was not supported by our analyses. An alternative hypothesis might be based on acculturation strategies, which also ought to depend strongly on integration policies for immigrants in a particular country. Our findings does not support a call for specific "culture sensitive" antismoking policies or interventions in Sweden or similar countries, but reinforce the existing evidence that a focus should be set on women with a low educational level, regardless of cultural background.

## Competing interests

The authors declare that they have no competing interests.

## Authors' contributions

KM led the writing and completed the analyses. P-OO, FE and AK helped in conceiving the study, conceptualising ideas, interpreting the results, and reviewing drafts of the article. All authors read and approved the final manuscript.

## Pre-publication history

The pre-publication history for this paper can be accessed here:

http://www.biomedcentral.com/1471-2458/10/374/prepub
